# Measurement of the morphological data of primary teeth in northwest China

**DOI:** 10.3389/fped.2022.1010423

**Published:** 2022-12-02

**Authors:** Xiao-Xi Lu, Kuan Yang, Bai-Ze Zhang, Jun-Hui Wang, Yang Du, Yu-Jiang Chen, Xiao-Jing Wang

**Affiliations:** ^1^ State Key Laboratory of Military Stomatology, National Clinical Research Center for Oral Diseases, Shaanxi Key Laboratory of Stomatology, Department of Pediatric Dentistry, School of Stomatology, Fourth Military Medical University, Xi’an, China; ^2^ Department of Orthodontics, Affiliated Hospital of Qingdao University, School of Stomatology, Qingdao University, Qingdao, China

**Keywords:** callipers, intraoral scanner, CBCT image measurement, primary teeth, northwest China

## Abstract

**Objective:**

This study aims to digitally obtain the morphological data of children's primary teeth in northwest China and evaluate the reliability of digitally obtaining the anatomical morphological data of primary teeth.

**Methods:**

A total of 308 extracted primary teeth and cone-beam computed tomography (CBCT) images of 407 primary teeth were collected in northwest China. Electronic digital Vernier callipers (accuracy: 0.01 mm) were used to measure the mesiodistal and buccolingual diameters and crown length of the extracted primary teeth and calculate the crown area and crown index. Each sample was scanned with an intraoral scanner (Trios2 3shape, Denmark), and the resulting stl format files were imported into Geomagic Wrap 2015 to measure the axial and buccolingual diameters and crown length. The crown area and crown index were then calculated. After verifying the accuracy of the CBCT image measurement, the CBCT image data of 407 samples were measured in SmartV software using the “measure length” function by referring to the coronal, sagittal, and horizontal planes to adjust the position of the reference line.

**Results:**

Northern Chinese have larger primary teeth than other populations (Japanese, white American, African, Icelander, Spanish, and Dominican Mestizo) but smaller primary teeth than native Australians. Compared to Indian primary teeth, northwest Chinese's primary teeth have larger diameters on the central axis and smaller diameters on the buccolingual surface. Male teeth are usually larger than female teeth. Compared with the results of Wang Huiyun's study, the axial and buccolingual diameters and crown length of all native tooth types in this total sample were significantly smaller at the 0.1% level, and only the axial diameters of the upper first molar and lower second molar and the crown length of the lower lateral incisor were significantly smaller at the 1% level. The results of the intraclass correlation coefficient of 308 extracted primary teeth expressed an excellent degree of agreement between the callipers and intraoral scanner for the following: mesiodistal diameter (0.956–0.991), buccolingual diameter (0.963–0.989), crown length [0.864–0.992, except for the upper canine (0.690)], crown index (0.850–0.975), and crown area (0.946–0.993).

**Conclusion:**

The digital measurements of the intraoral scanner and CBCT image are in good agreement with the manual measurement of the Vernier calliper. The difference between the anatomical morphology size of the primary teeth measured in this study and the results of different populations could be due to different genetic backgrounds and environmental factors.

## Introduction

The morphological data of primary teeth in children provide an important reference for dental restorative treatment, growth and development research, crowding prediction, and treatment of malocclusion. Some predictions of tooth growth in adults can be made through dental data of children (1, 2). According to the teeth number, size, and shape changes, some dental problems can be diagnosed early, resulting in the best patient management and treatment plan. Moreover, intervention can be performed at the appropriate time to prevent complications and reduce later treatment needs. For example, the extraction of supernumerary teeth would otherwise interfere with the eruption of the underlying permanent teeth (3). Collecting data on some native teeth can predict the condition of other teeth; for example, unerupted maxillary and mandibular canines and premolars can be assessed with the mesiodistal width of erupted mandibular permanent incisors, and prediction of the size of unerupted teeth in mixed dentition is a key factor when formulating treatment plans and dealing with malformations (4). Abnormal tooth size is an important cause of malocclusion. To obtain a good orthodontic effect of malocclusion, the size and proportion of maxillary and mandibular teeth need to be coordinated, and the collected data can provide a reference for its treatment (5). Gender dimorphism in deciduous crown size is often used to identify the sex of subadults from archaeological sites and forensic settings because sex identification by measuring tooth anatomy is relatively highly accurate (6, 7). For the measurement of the morphology of primary teeth, scholars from various countries use manual measurement methods represented by Vernier callipers to measure the primary teeth and established reference values for the morphological data of primary teeth in this area (8–10). In China, Professor Wang Huiyun has established a reference standard value for the morphological data of Chinese teeth by measuring more than 9,000 extracted teeth in the 1950s. However, the number of samples of primary teeth is extremely limited, and the samples are only from east China (11). So far, there is no literature on the morphological data of children's primary teeth in northwest China.

With the advent of the digital age, three-dimensional measurement technology is fully developed in the field of stomatology due to its accuracy and accessibility (12), and it has relatively mature applications in the measurement of tooth morphology (13, 14). However, there are no studies on the three-dimensional digital measurement of primary teeth morphology. The purpose of this study is to digitally obtain the morphological data of children's primary teeth in northwest China and evaluate the reliability of digitally obtaining the anatomical morphological data of primary teeth.

## Materials and methods

### Data measurement of extracted primary teeth

In this study, 308 paediatric stomatology patients who were treated in the Department of Pediatric Stomatology of the Third Affiliated Hospital of Air Force Military Medical University from July 2020 to June 2021 were selected by convenience sampling for a retrospective study. Each patient had one tooth, giving a total of 308 patients. The inclusion criteria were as follows: (1) extracted due to factors such as retention and trauma; (2) intact crowns; and (3) the subjects were willing to comply with all the study procedures and protocols. The exclusion criteria were as follows: (1) obvious caries; (2) abnormal tooth development; (3) systemic diseases; and (4) a medical history with conditions that could affect oral health. Informed consent was obtained from the patients and their families for this study. This study was approved by the Ethics Review Institution of the Ethics Committee of Fourth Military Medical University, which complied with the Declaration of Helsinki. Informed consent was obtained from all participants for inclusion in the study.

**Figure 1:**
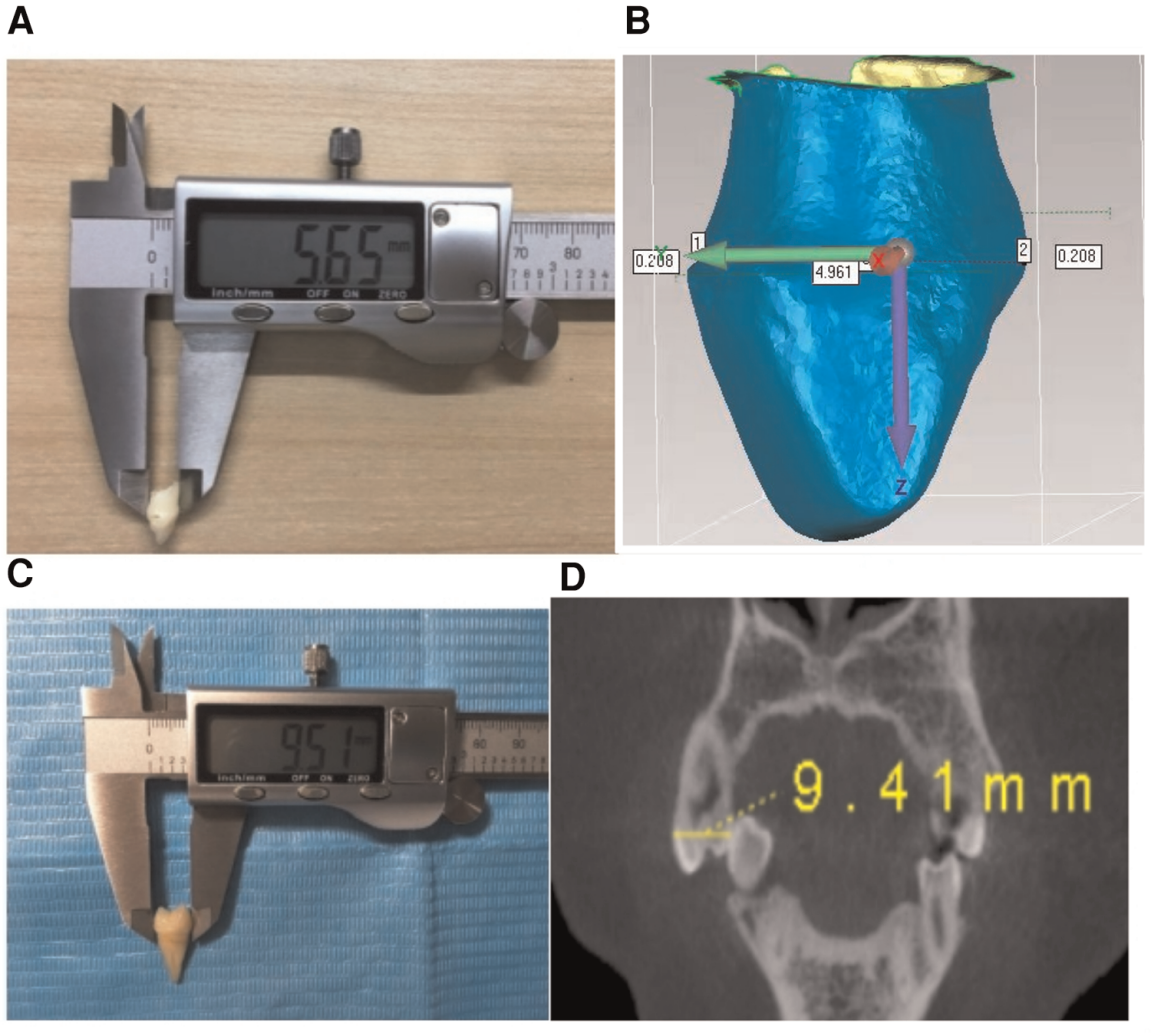
(**A**) Manual measurement of extracted primary teeth with a Vernier calliper. (**B)** Digital measurement of extracted primary teeth with an intraoral scanner. (**C**) Manual measurement of extracted maxillary first premolar with a Vernier calliper. (**D**) CBCT image digital measurement of maxillary first premolars.

We put the extracted primary teeth that met the inclusion criteria into a 10% formaldehyde solution and removed the tissues and blood stains. When handling the teeth, we avoided damaging the tooth tissue and used 2% glutaraldehyde to disinfect it. We dried the tooth before measurement. We used electronic digital Vernier callipers (Deli DL911504, accuracy 0.01 mm) to measure the mesiodistal and buccolingual diameters and crown length of the extracted primary teeth that met the inclusion criteria and calculated the crown area and crown index. Each measurement item was measured three times by the same researcher, and the average value was taken (Figure 1A). Then, each sample was scanned with an intraoral scanner (Trios2 3shape, Denmark), and the generated stl format file was imported into Geomagic Wrap 2015 (Geomagic, United States). The distance measurement function of the software was used to measure the mesiodistal and buccolingual diameters and crown length. Then, the crown area and crown index were calculated. Each measurement item was measured three times by the same researcher, and the average value was taken (Figure 1B).

### Annotation of measurement items

Mesiodistal diameter: The vertical distance between the mesial and distal contact points of the crown.

Buccolingual diameter: The vertical distance between the most protruding point of the labial or buccal surface and the most protruding point of the lingual surface of the crown.

Crown length: The vertical distance from the incisal edge or cusp (the longest cusp on the crown) to the most apical point on the labial or buccal cervical edge of the crown.

Crown index: (buccolingual diameter × 100)/mesiodistal diameter.

Crown area: mesiodistal diameter × buccolingual diameter.

### Data measurement of primary teeth from cone-beam computed tomography

#### Research on the accuracy of cone-beam computed tomography imaging measurement

A total of 50 extracted maxillary first premolars were selected from July 2020 to September 2020 at the Maxillofacial Surgery Clinic of the Third Affiliated Hospital of Air Force Medical University. Each patient had one tooth, giving a total of 50 patients. The apex development was complete with no caries, fillings, or history of endodontic treatment, and the pre-measurement treatment was the same as that of the extracted primary teeth. Electronic digital Vernier callipers (Deli DL911504, accuracy 0.01 mm) were used to measure the mesiodistal and buccolingual diameters and crown length of the 50 samples (Figure 1C). Each measurement item was measured three times by the same researcher, and the average value was taken. The cone-beam computed tomography (CBCT) image data of the 50 samples were collected from the Radiology Department of the Third Affiliated Hospital of the Air Force Military Medical University, and their mesiodistal and buccolingual diameters and crown length were measured using SmartV software (Smart V 2.0, Beijing Langshi Instruments Co., Ltd., China) using the “measure length” function by referring to the coronal, sagittal ,and horizontal planes to adjust the position of the reference line (Figure 1D). Each measurement item was measured three times by the same researcher, and the average value was taken. The consistency analysis, paired *t*-test, and regression analysis of the data measured by the two measurement methods were carried out.

#### CBCT imaging measurement of primary teeth

After verifying the accuracy of the CBCT image measurement, the CBCT image data of children at primary and mixed dentition stages from July 2020 to June 2021 in the Radiology Department of the Third Affiliated Hospital of Air Force Medical University were selected (CBCT HiRes3D, Beijing Longche). The shooting parameters were as follows: voltage: 100 kV, current: 4 mA, visual field size: 8 cm × 15 cm, and voxel: 0.25 mm. A total of 407 primary teeth with complete crowns and no obvious caries or abnormal tooth development were collected. The mesiodistal and buccolingual diameters and crown length were measured using SmartV software using the “measure length” function by referring to the coronal, sagittal, and horizontal planes to adjust the position of the reference line, and the crown area and crown index were calculated. Each measurement item was measured three times by the same researcher, and the average value was taken.

### Statistical analysis

A sexual dimorphism analysis was performed on all measured items for all samples as follows: sexual dimorphism = (male mean − female mean)/female mean × 100%. SPSS 26.0 was used for the analysis. Measurement data conforming to normal distribution were expressed as the mean. Manual measurements with Vernier callipers and digital measurements with intraoral scanners were performed on 308 extracted deciduous teeth. Manual measurements with Vernier callipers and CBCT measurements on 50 maxillary first premolars were first subjected to a paired *t*-test, and *P* < 0.05 indicated statistical significance. Correlation and regression analyses were then performed to determine whether there was a correlation and regression relationship based on the correlation coefficient and coefficient of determination compared within the group.

## Results

The results of the paired *t*-test and consistency and regression analyses of 308 extracted primary teeth by manual and digital measurements are shown in Tables 1, 2. The results of the paired *t*-test showed that there was no significant difference in the results of the two measurement methods in 96% of the measurement items (*P* > 0.05). The results of the consistency and regression analyses showed that except for the crown length of upper canine (UC) [intraclass correlation coefficient (ICC)* *= 0.690, *R*^2^ = 0.468], the ICC and determination coefficient (*R*^2^) of each measurement item in the remaining tooth type were higher (ICC: 0.850–0.993, *R*^2^: 0.726–0.986), indicating that there is high consistency between the manual measurement with Vernier callipers and digital measurement with an intraoral scanner.

**Table 1:** Results of paired *t*-test, consistency analysis, and regression analysis for mesiodistal diameter buccolingual diameter and crown length of 308 extracted primary teeth by manual and digital measurement.

Tooth type	*N*	Mesiodistal diameter			Buccolingual diameter			Crown length		
		*P*	ICC	*R* ^2^	*P*	ICC	*R* ^2^	*P*	ICC	*R* ^2^
UA	45	0.080	0.991	0.985	0.381	0.98	0.962	0.136	0.988	0.977
UB	21	0.338	0.983	0.969	0.152	0.979	0.97	0.565	0.988	0.976
UC	24	0.104	0.967	0.956	0.261	0.985	0.973	0.020	0.690	0.468
UD	22	0.815	0.984	0.967	0.500	0.988	0.977	0.545	0.978	0.955
UE	25	0.968	0.965	0.931	0.163	0.985	0.974	0.186	0.982	0.966
LA	80	0.597	0.984	0.968	0.279	0.968	0.942	0.304	0.973	0.948
LB	22	0.694	0.989	0.94	0.820	0.989	0.977	0.754	0.992	0.985
LC	19	0.308	0.968	0.952	0.832	0.977	0.954	0.042	0.985	0.972
LD	21	0.439	0.987	0.980	0.230	0.989	0.985	0.198	0.962	0.937
LE	29	0.855	0.989	0.978	0.688	0.963	0.927	0.244	0.864	0.882

ICC, intraclass correlation coefficient; UA, upper central incisor; UB, upper lateral incisor; UC, upper canine; UD, upper first molar; UE, upper second molar; LA, lower central incisor; LB, lower lateral incisor; LC, lower canine; LD, lower first molar; LE, lower second molar.

**Table 2:** Results of paired *t*-test, consistency analysis, and regression analysis for crown index and crown area of 308 extracted primary teeth by manual and digital measurement.

Tooth type	*N*	Crown index			Crown area		
		*P*	ICC	*R* ^2^	*P*	ICC	*R* ^2^
UA	45	0.215	0.943	0.897	0.205	0.946	0.922
UB	21	0. 203	0.96	0.954	0.429	0.983	0.971
UC	24	0.135	0.871	0.799	0.432	0.989	0.986
UD	22	0.745	0.947	0.895	0.732	0.993	0.986
UE	25	0.334	0.85	0.726	0.891	0.978	0.955
LA	80	0. 112	0.947	0.904	0.317	0.968	0.94
LB	22	0.851	0.967	0.938	0.468	0.988	0.976
LC	19	0.151	0.929	0.871	0.552	0.981	0.962
LD	21	0.901	0.975	0.964	0.112	0.992	0.986
LE	29	0.249	0.962	0.93	0.648	0.984	0.97

ICC, intraclass correlation coefficient; UA, upper central incisor; UB, upper lateral incisor; UC, upper canine; UD, upper first molar; UE, upper second molar; LA, lower central incisor; LB, lower lateral incisor; LC, lower canine; LD, lower first molar; LE, lower second molar.

The results of the paired *t*-test and consistency and regression analyses of 50 extracted maxillary first premolars by manual and CBCT image measurement are shown in Table 3. The results of the paired *t*-test show that there is no statistically significant difference in the results of the two measurement methods for the three measurement items of the 50 maxillary first premolars (*P* > 0.05). The value of the ICC and *R*^2^ of the two measurement methods for the three measurement items of the 50 maxillary first premolars are high, indicating that there is a high consistency between the manual measurement with Vernier callipers and digital measurement of the CBCT image.

**Table 3:** Results of paired *t*-test, consistency analysis, and regression analysis for each measurement item of 50 maxillary first premolars by manual and CBCT image measurement.

Measurement item	*t* value	*P* value	ICC	*R* ^2^
Mesiodistal diameter	0.847	0.401	0.959	0.920
Buccolingual diameter	1.958	0.056	0.997	0.995
Crown length	1.444	0.155	0.999	0.998

ICC, intraclass correlation coefficient.

**Figure 2:**
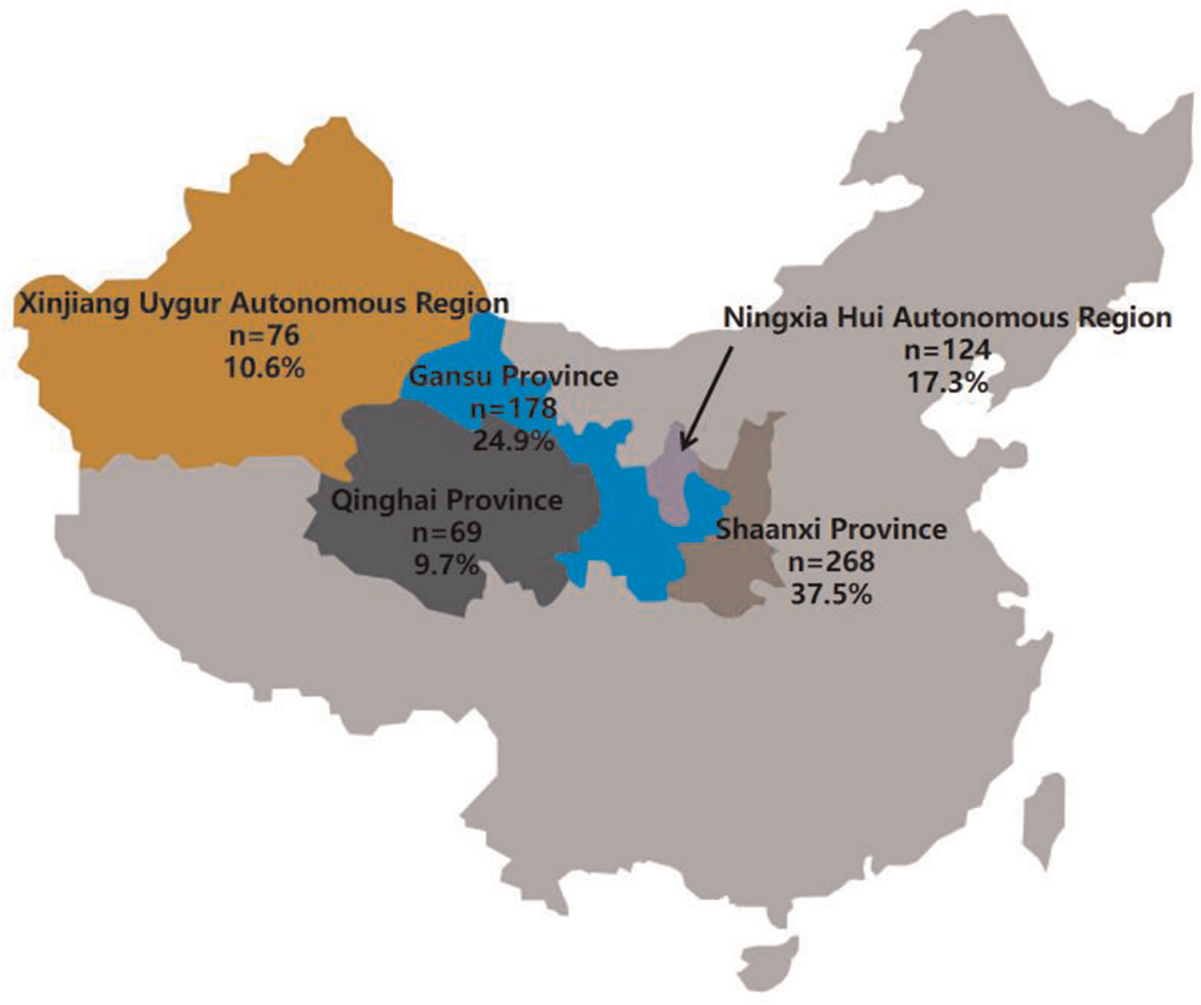
Geographical distribution of the total sample with a sample size of 715 collected from CBCT images of primary teeth and extracted primary teeth.

After confirming that the digital measurements of the intraoral scanner and CBCT image were in good agreement with the manual measurement of the Vernier calliper, the digital measurement data of the intraoral scanner of 308 extracted primary teeth and the CBCT image data of 407 primary teeth were summarised. The geographical source of the total sample is shown in Figure 2. Of the total sample, 37.5% are from the Shaanxi Province, 24.9% are from the Gansu Province, 17.3% are from the Ningxia Hui Autonomous Region, 10.6% are from the Xinjiang Uygur Autonomous Region, and 9.7% are from the Qinghai Province.

The results of all measurement items for all samples showed that the central axis diameter of the four main tooth types was significantly larger than that of females at the 0.1% level (Tables 4–8). The mesiodistal diameter of one primary tooth type was significantly larger than that of females at the 1% level. The mesiodistal diameters of two primary tooth types were significantly larger than that of females at the 5% level. The buccolingual diameters of two primary tooth types were significantly larger than that of females at the 0.1% level. The buccolingual diameters of two primary tooth types were significantly larger than that of females at the 1% level. The crown length of one primary tooth type was significantly larger than that of females at the 0.1% level. The crown lengths of two primary tooth types were significantly larger than that of females at the 1% level. The crown length of one primary tooth type was significantly larger than that of females at the 5% level. The crown area of five primary tooth types was significantly larger than that of females at the 0.1% level. The crown area of one primary tooth type was significantly larger than that of females at the 1% level. The crown areas of two primary tooth types were significantly larger than that of females at the 5% level. The sexual dimorphism percentage of each measurement items are as follows: mesiodistal diameter (0.59–2.54), buccolingual diameter (0.80–2.74), crown length (0.83–2.72), crown index (−0.71 to 2.48), and crown area (1.41–10.65).

**Table 4:** Mesiodistal diameter (mm) and sexual dimorphism of the total sample.

Tooth type	Sex	*N*	Mesiodistal diameter				
			Mean	SD	CV	Difference (M − F)	Sexual dimorphism %
UA	M	36	6.83	0.35	5.12	0.04	0.59
	F	22	6.79	0.38	5.59		
UB	M	26	5.64	0.20	3.55	0.07	1.26
	F	15	5.57	0.31	5.57		
UC	M	48	7.20	0.35	4.86	0.13***	1.84
	F	39	7.07	0.14	1.98		
UD	M	40	7.36	0.28	3.80	0.10*	1.38
	F	30	7.26	0.22	3.03		
UE	M	53	9.37	0.24	2.56	0.20***	2.18
	F	39	9.17	0.24	2.62		
LA	M	51	4.27	0.21	4.92	0.10*	2.40
	F	39	4.17	0.22	5.28		
LB	M	16	5.07	0.23	4.54	0.12	2.4
	F	19	4.95	0.27	5.45		
LC	M	41	6.05	0.16	2.64	0.15***	2.54
	F	29	5.90	0.22	3.73		
LD	M	43	8.38	0.15	1.79	0.20***	2.44
	F	32	8.18	0.25	3.06		
LE	M	61	10.49	0.19	1.81	0.15**	1.45
	F	36	10.34	0.29	2.80		

UA, upper central incisor; UB, upper lateral incisor; UC, upper canine; UD, upper first molar; UE, upper second molar; LA, lower central incisor; LB, lower lateral incisor; LC, lower canine; LD, lower first molar; LE, lower second molar. SD, standard deviation; CV, coefficient of variation.

* *P* < 0.05; **P < 0.01; ***P < 0.001.

**Table 5:** Buccolingual diameter (mm) and sexual dimorphism of the total sample.

Tooth type	Sex	*N*	Buccolingual diameter				
			Mean	SD	CV	Difference (M − F)	Sexual dimorphism %
UA	M	36	5.01	0.35	6.99	0.04	0.80
	F	22	4.97	0.22	4.43		
UB	M	26	5.26	0.24	4.56	0.05	0.96
	F	15	5.21	0.30	5.76		
UC	M	48	6.07	0.17	2.80	0.08	1.34
	F	39	5.99	0.19	3.17		
UD	M	40	9.10	0.35	3.85	0.14	1.56
	F	30	8.96	0.21	2.34		
UE	M	53	10.03	0.23	2.29	0.16***	1.62
	F	39	9.87	0.26	2.63		
LA	M	51	3.83	0.25	6.53	0.08	2.13
	F	39	3.75	0.25	6.67		
LB	M	16	4.53	0.22	4.86	0.04	0.89
	F	19	4.49	0.30	6.68		
LC	M	41	5.69	0.32	5.62	0.13**	2.34
	F	29	5.56	0.25	4.49		
LD	M	43	7.51	0.20	2.66	0.20**	2.74
	F	32	7.31	0.29	3.97		
LE	M	61	9.30	0.21	2.26	0.19***	2.09
	F	36	9.11	0.24	2.63		

UA, upper central incisor; UB, upper lateral incisor; UC, upper canine; UD, upper first molar; UE, upper second molar; LA, lower central incisor; LB, lower lateral incisor; LC, lower canine; LD, lower first molar; LE, lower second molar. SD, standard deviation; CV, coefficient of variation.

* *P* < 0.05; **P < 0.01; ***P < 0.001.

**Table 6:** Crown length (mm) and sexual dimorphism of the total sample.

Tooth type	Sex	*N*	Crown length				
			Mean	SD	CV	Difference (M − F)	Sexual dimorphism %
UA	M	36	6.45	0.36	5.58	0.16	2.54
	F	22	6.29	0.35	5.56		
UB	M	26	6.34	0.34	5.36	0.10	1.60
	F	15	6.24	0.20	3.21		
UC	M	48	6.84	0.25	3.65	0.07*	1.03
	F	39	6.77	0.30	4.43		
UD	M	40	6.26	0.25	3.99	0.08	1.29
	F	30	6.18	0.16	2.59		
UE	M	53	6.79	0.16	2.36	0.15**	2.26
	F	39	6.64	0.26	3.92		
LA	M	51	5.67	0.49	8.64	0.15	2.72
	F	39	5.52	0.47	8.51		
LB	M	16	6.39	0.33	5.16	0.16	2.57
	F	19	6.23	0.36	5.78		
LC	M	41	7.30	0.22	3.01	0.06	0.83
	F	29	7.24	0.24	3.31		
LD	M	43	7.03	0.19	2.70	0.14**	2.03
	F	32	6.89	0.26	3.77		
LE	M	61	6.83	0.21	3.07	0.15***	2.25
	F	36	6.68	0.18	2.69		

UA, upper central incisor; UB, upper lateral incisor; UC, upper canine; UD, upper first molar; UE, upper second molar; LA, lower central incisor; LB, lower lateral incisor; LC, lower canine; LD, lower first molar; LE, lower second molar. SD, standard deviation; CV, coefficient of variation.

* *P* < 0.05; **P < 0.01; ***P < 0.001.

**Table 7:** Crown index and sexual dimorphism of the total sample.

Tooth type	Sex	*N*	Crown index				
			Mean	SD	CV	Difference (M − F)	Sexual dimorphism %
UA	M	36	72.32	3.65	5.05	0.13	0.18
	F	22	72.19	2.62	3.63		
UB	M	26	92.30	2.47	2.67	0.24	0.26
	F	15	92.06	4.93	5.36		
UC	M	48	85.06	2.32	2.73	0.29	0.34
	F	39	84.77	1.72	2.03		
UD	M	40	123.63	2.21	1.79	0.15	0.12
	F	30	123.48	3.28	2.65		
UE	M	53	107.13	1.37	1.28	−0.53	−0.49
	F	39	107.66	1.62	1.50		
LA	M	51	89.47	5.26	5.88	−0.64	−0.71
	F	39	90.11	5.28	5.86		
LB	M	16	91.70	3.07	3.35	2.22	2.48
	F	19	89.48	4.94	5.52		
LC	M	41	94.08	2.51	2.67	−0.07	−0.07
	F	29	94.15	2.59	2.75		
LD	M	43	90.74	1.62	1.79	1.38	1.54
	F	32	89.36	4.54	5.08		
LE	M	61	88.30	3.31	3.75	0.07	0.08
	F	36	88.23	1.39	1.57		

UA, upper central incisor; UB, upper lateral incisor; UC, upper canine; UD, upper first molar; UE, upper second molar; LA, lower central incisor; LB, lower lateral incisor; LC, lower canine; LD, lower first molar; LE, lower second molar. SD, standard deviation; CV, coefficient of variation.

* *P* < 0.05; **P < 0.01; ***P < 0.001.

**Table 8:** Crown area and sexual dimorphism of the total sample.

Tooth type	Sex	*N*	Crown area				
			Mean	SD	CV	Difference (M − F)	Sexual dimorphism %
UA	M	36	34.41	4.30	12.49	0.48	1.41
	F	22	33.93	3.92	11.55		
UB	M	26	31.53	2.69	8.53	2.89**	10.09
	F	15	28.64	3.06	10.68		
UC	M	48	44.09	1.49	3.38	2.43***	5.83
	F	39	41.66	2.83	6.79		
UD	M	40	67.04	4.39	6.54	1.28	1.95
	F	30	65.76	4.50	6.84		
UE	M	53	94.05	2.93	3.11	3.50***	3.87
	F	39	90.55	4.00	4.42		
LA	M	51	16.85	2.22	13.17	1.04*	6.58
	F	39	15.81	1.85	11.70		
LB	M	16	24.41	2.27	9.29	2.35*	10.65
	F	19	22.06	2.72	12.33		
LC	M	41	34.49	1.70	4.93	1.58***	4.80
	F	29	32.91	1.56	4.74		
LD	M	43	63.70	2.22	3.49	3.91***	6.54
	F	32	59.79	3.73	6.24		
LE	M	61	97.57	2.15	2.20	3.40***	3.61
	F	36	94.17	4.48	4.76		

UA, upper central incisor; UB, upper lateral incisor; UC, upper canine; UD, upper first molar; UE, upper second molar; LA, lower central incisor; LB, lower lateral incisor; LC, lower canine; LD, lower first molar; LE, lower second molar. SD, standard deviation; CV, coefficient of variation.

* *P* < 0.05; **P < 0.01; ***P < 0.001.

The comparisons between the measurement results of each measurement item of the total sample (except crown length) and the measurement results of other populations are shown in Tables 9–12.

**Table 9:** Difference (mm) between the mesiodistal diameter of the primary teeth of northwest Chinese and those of the other populations.

Tooth type	Japanese	Australian Aboriginal	Indian	American White	African American	Icelander	Spanish	Dominican Mulatto
Male								
UA	0.08	−0.52***	0.00	0.28***	0.13*	0.34***	0.35***	0.22**
UB	0.14**	−0.36***	0.09***	0.32***	0.24***	0.29***	0.72***	0.41***
UC	0.44***	−0.21***	0.40***	0.32***	0.30***	0.22***	0.21***	0.63***
UD	−0.14***	−0.19***	−0.02	0.24***	−0.14***	0.19***	−0.16***	0.10**
UE	0.13***	−0.28***	0.37***	0.29***	0.17***	0.37***	0.59***	−0.41***
LA	−0.01	−0.24***	−0.06*	0.19***	0.07*	0.00	0.41***	0.19***
LB	0.22**	0.06	0.17*	0.33***	0.37***	0.37***	0.71***	0.46***
LC	0.22***	−0.26***	0.15***	0.13***	0.05**	0.11***	0.06**	0.37***
LD	0.04*	0.13***	0.00	0.58***	0.18***	0.40***	0.31***	0.43***
LE	0.23***	−0.40***	0.36***	0.66***	0.19***	0.38***	0.42***	0.56***
Female								
UA	0.19*	−0.41***	0.24**	0.35***	0.29***	0.36***	0.38***	0.30***
UB	0.21**	−0.36***	0.22**	0.34***	0.27**	0.29***	0.81***	0.32***
UC	0.40***	−0.14***	0.42***	0.40***	0.37***	0.17***	0.01	0.55***
UD	−0.03	−0.02	0.01	0.31***	0.06	0.22***	0.05	0.12**
UE	0.07	−0.25***	0.32***	0.33***	0.37***	0.20***	0.42***	−0.48***
LA	−0.01	−0.17***	−0.02	0.19***	0.17***	0.27***	0.39***	0.23***
LB	0.20**	0.04	0.22**	0.32***	0.35***	0.38***	0.61***	0.41***
LC	0.05	−0.26***	0.05	0.16***	0.10*	0.08	−0.29***	0.26***
LD	0.02	0.06	0.18***	0.53***	0.28***	0.37***	0.33***	0.29***
LE	0.20***	−0.30***	0.39***	0.70***	0.44***	0.39***	0.56***	0.57***

UA, upper central incisor; UB, upper lateral incisor; UC, upper canine; UD, upper first molar; UE, upper second molar; LA, lower central incisor; LB, lower lateral incisor; LC, lower canine; LD, lower first molar; LE, lower second molar.

* *P* < 0.05; **P < 0.01; ***P < 0.001.

**Table 10:** Difference (mm) between the buccolingual diameter of the primary teeth of northwest Chinese and those of the other populations.

Tooth type	Australian Aboriginal	Indian	Icelander	Spanish	Dominican Mulatto
Male					
UA	−0.46***	−0.39***	−0.07	−0.08	0.00
UB	0.02	0.41***	0.25***	0.52***	0.61***
UC	−0.54***	−0.03	−0.30***	−0.19***	0.13***
UD	0.03	0.30***	0.23***	0.43***	0.52***
UE	−0.62***	−0.07**	−0.07**	0.17***	0.25***
LA	−0.50***	−0.37***	−0.08*	0.20***	0.18***
LB	−0.22**	−0.12*	0.08	0.50***	0.45***
LC	−0.36***	−0.01	−0.02	0.14***	0.39***
LD	−0.41***	−0.24***	0.16***	−0.11**	0.30***
LE	−0.57***	−0.28***	0.21***	0.63***	0.43***
Female					
UA	−0.33***	−0.26***	−0.04	0.20***	−0.03
UB	0.20*	0.63***	0.28**	0.65***	0.46***
UC	−0.35***	0.19***	−0.28***	−0.35***	−0.13***
UD	0.19***	0.31***	0.27***	0.46***	0.33***
UE	−0.40***	−0.08*	−0.01	0.47***	0.05
LA	−0.44***	−0.33***	−0.03	0.15***	0.01
LB	−0.16*	−0.06	0.20**	0.45***	0.32***
LC	−0.28***	0.08**	−0.04	−0.11***	0.04
LD	−0.18**	−0.07	0.02	−0.12	0.04
LE	−0.46***	−0.19***	0.09**	0.79***	0.10**

UA, upper central incisor; UB, upper lateral incisor; UC, upper canine; UD, upper first molar; UE, upper second molar; LA, lower central incisor; LB, lower lateral incisor; LC, lower canine; LD, lower first molar; LE, lower second molar.

* *P* < 0.05; **P < 0.01; ***P < 0.001.

**Table 11:** Difference between the crown index of the primary teeth of northwest Chinese and those of the other populations.

Tooth type	Australian Aboriginal	Icelander	Dominican Mulatto
Male			
UA	−2.21**	−5.36***	−3.89***
UB	5.15***	−1.63**	3.90***
UC	−4.19***	−6.47***	−5.89***
UD	3.00***	−0.46	4.94***
UE	−3.39***	−5.25***	−4.75***
LA	−6.86***	−1.87*	0.72
LB	−3.43***	−2.99***	3.71***
LC	−2.16***	−2.52***	−0.29
LD	−5.43***	−0.73**	1.01***
LE	−2.36***	−1.37**	−1.41**
Female			
UA	−1.30*	−5.90***	−5.50***
UB	7.54***	−1.39	1.20
UC	−3.19***	−6.27***	−9.37***
UD	2.69***	−0.04	2.59***
UE	−1.43***	−2.75***	−3.05***
LA	−6.68***	−7.60***	−4.63***
LB	−5.80***	−5.07***	−2.87*
LC	−1.28*	−2.45***	−4.06***
LD	−2.91**	−3.69***	−2.72**
LE	−1.68***	−2.22***	−3.57***

UA, upper central incisor; UB, upper lateral incisor; UC, upper canine; UD, upper first molar; UE, upper second molar; LA, lower central incisor; LB, lower lateral incisor; LC, lower canine; LD, lower first molar; LE, lower second molar.

* *P* < 0.05; **P < 0.01; ***P < 0.001.

**Table 12:** Difference between the crown area of the primary teeth of northwest Chinese and those of the other populations.

Tooth type	Australian Aboriginal	Icelander	Dominican Mulatto
Male			
UA	−5.68***	0.92	1.00
UB	0.23	4.53***	6.78***
UC	−4.67***	−0.34	4.58***
UD	−1.28	3.36***	4.37***
UE	−8.58***	3.00***	7.45***
LA	−2.62***	0.08	1.97***
LB	0.71	3.37***	5.52***
LC	−3.72***	0.47	4.05***
LD	−1.49***	5.04***	5.03***
LE	−9.70***	5.70***	8.52***
Female			
UA	−4.06***	1.62	1.18
UB	−0.96	2.36*	3.45**
UC	−3.90***	−1.68**	1.38**
UD	2.11**	4.29***	4.62***
UE	−5.81***	1.71*	4.79***
LA	−2.43***	0.95**	0.81*
LB	−0.58	2.41**	2.96***
LC	−2.91***	0.23	1.16***
LD	−0.87	3.05***	1.61*
LE	−7.25***	4.40***	5.93***

UA, upper central incisor; UB, upper lateral incisor; UC, upper canine; UD, upper first molar; UE, upper second molar; LA, lower central incisor; LB, lower lateral incisor; LC, lower canine; LD, lower first molar; LE, lower second molar.

* *P* < 0.05; **P < 0.01; ***P < 0.001.

When compared with the Japanese population (2), four primary teeth in males and two primary teeth in females were significantly larger in northwest Chinese at the 0.1% level, two primary teeth in males and two primary teeth in females were significantly larger at the 1% level, and one primary tooth in males and one primary tooth in females were significantly larger at the 5% level. When compared with the Australian Aboriginal population (15), eight primary teeth in males and seven primary teeth in females were significantly smaller in northwest Chinese at the 0.1% level. When compared with the Indian population (16), four primary teeth in males and four primary teeth in females were significantly larger in northwest Chinese at the 0.1% level, three primary teeth in females were significantly larger at the 1% level, and two primary teeth in males were significantly larger at the 5% level. When compared with the American white population (17), all 10 primary teeth both in males and females were significantly larger in northwest Chinese at the 0.1% level. When compared with the African American population (18), six primary teeth in males and seven primary teeth in females were significantly larger in northwest Chinese at the 0.1% level, one primary tooth in males and one primary tooth in females were significantly larger at the 1% level, and two primary teeth in males and one primary tooth in females were significantly larger at the 5% level. When compared with the Icelander population (19), nine primary teeth in males and nine primary teeth in females were significantly larger in northwest Chinese at the 0.1% level. When compared with the Spanish population (20), eight primary teeth in males and seven primary teeth in females were significantly larger in northwest Chinese at the 0.1% level, and one primary tooth in males was significantly larger at the 1% level. When compared with the Dominican Mulatto population (21), eight primary teeth in males and eight primary teeth in females were significantly larger in northwest Chinese at the 0.1% level, and one primary tooth in males and one primary tooth in females were significantly larger at the 1% level.

When compared with the Australian Aboriginal population (15), seven primary teeth in males and six primary teeth in females were significantly smaller in northwest Chinese at the 0.1% level, one primary tooth in males and one primary tooth in females were significantly smaller at the 1% level, and one primary tooth in females was significantly smaller at the 5% level. When compared with the Indian population (16), four primary teeth in males and three primary teeth in females were significantly smaller in northwest Chinese at the 0.1% level, one primary tooth in males was significantly smaller at the 1% level, and one primary tooth in males and one primary tooth in females were significantly smaller at the 5% level. When compared with the Icelander population (19), four primary teeth in males and two primary teeth in females were significantly larger in northwest Chinese at the 0.1% level, two primary teeth in females were significantly larger at the 1% level, one primary tooth in males and one primary tooth in females were significantly smaller at the 0.1% level, one primary tooth in males was significantly smaller at the 1% level. and one primary tooth in males was significantly smaller at the 5% level. When compared with the Spanish population (20), seven primary teeth in males and seven primary teeth in females were significantly larger in northwest Chinese at the 0.1% level. When compared with the Dominican Mulatto population (21), eight primary teeth in males and three primary teeth in females were significantly larger in northwest Chinese at the 0.1% level, and one primary tooth in females was significantly larger at the 1% level.

When compared with the Australian Aboriginal population (15), seven primary teeth in males and five primary teeth in females were significantly smaller in northwest Chinese at the 0.1% level, one primary tooth in males and one primary tooth in females were significantly smaller at the 1% level, and two primary teeth in females were significantly smaller at the 5% level. When compared with the Icelander population (19), five primary teeth in males and eight primary teeth in females were significantly smaller in northwest Chinese at the 0.1% level, three primary teeth in males were significantly smaller at the 1% level, and one primary tooth in males was significantly smaller at the 5% level. When compared with the Dominican Mulatto population (22), three primary teeth in males and six primary teeth in females were significantly smaller in northwest Chinese at the 0.1% level, one primary tooth in males and one primary tooth in females were significantly smaller at the 1% level, one primary tooth in females was significantly smaller at the 5% level, and four primary teeth in males were significantly larger at the 0.1% level.

When compared with the Australian Aboriginal population (15), seven primary teeth in males and six primary teeth in females were significantly smaller in northwest Chinese at the 0.1% level. When compared with the Icelander population (19), six primary teeth in males and three primary teeth in females were significantly larger in northwest Chinese at the 0.1% level, two primary teeth in females were significantly larger at the 1% level, and two primary teeth in females were significantly larger at the 5% level. When compared with the Dominican Mulatto population (22), nine primary teeth in males and five primary teeth in females were significantly larger in northwest Chinese at the 0.1% level, two primary teeth in females were significantly larger at the 1% level, and two primary teeth in females were significantly larger at the 5% level.

The comparisons between the measurement results of the mesiodistal and buccolingual diameters and crown length of the total sample in the present study and the results of Wang Huiyun's study (11) are shown in Table 13.

**Table 13:** Difference between mesiodistal diameter buccolingual diameter and crown length of the present total sample and the results of Wang Huiyun's study.

	Mesiodistal diameter	Buccolingual diameter	Crown length
UA	−0.48***	−0.41***	−0.41***
UB	−0.38***	−0.34***	−0.30***
UC	−0.16***	−0.16***	−0.190***
UD	−0.08**	−0.16***	−0.17***
UE	−0.12***	−0.05***	−0.17***
LA	−0.57***	−0.60***	−0.90***
LB	−0.29***	−0.39***	−0.20**
LC	−0.11***	−0.16***	−0.13***
LD	−0.11***	−0.28***	−0.13***
LE	−0.07**	−0.07***	−0.13***

UA, upper central incisor; UB, upper lateral incisor; UC, upper canine; UD, upper first molar; UE, upper second molar; LA, lower central incisor; LB, lower lateral incisor; LC, lower canine; LD, lower first molar; LE, lower second molar.

* *P* < 0.05; **P < 0.01; ***P < 0.001.

The results of Table 13 show that all the measured items of all tooth types of the present total sample are significantly smaller at the 0.1% level than that in Wang Huiyun's study except for the mesiodistal diameter of the upper first molar (UD) and lower second molar (LE) and the crown length of lower lateral incisor (LB), which are significantly smaller at the 1% level.

## Discussion

In this study, 308 extracted primary teeth and the CBCT images of 407 primary teeth of northwest Chinese were collected. Tooth dimensions can be obtained by measuring the mesiodistal and buccolingual crown diameters and crown length (23, 24). The crown index and crown area are often used to obtain the overall size of primary teeth, as they can provide useful summaries and comprehensive descriptions of the morphology of primary teeth (22).

An intraoral scanner is a commonly used instrument to obtain three-dimensional digital models, and its accuracy has been confirmed in the literature (25, 26). The results of the intraclass correlation coefficient of 308 extracted primary teeth expressed an excellent degree of agreement between the callipers and intraoral scanner for the following: mesiodistal diameter (0.956–0.991), buccolingual diameter (0.963–0.989), crown length [0.864–0.992, except for UC (0.690)], crown index (0.850–0.975), and crown area (0.946–0.993). These results are consistent with those obtained by other studies that also analysed dental diameters using callipers and intraoral scanners. Soto-Álvarez et al. (27) found a high degree of agreement of extracted dry teeth measurements (mesiodistal diameter: 0.959–0.993, buccolingual diameter: 0.981–0.998) between callipers and intraoral scanners. Another study (28) also demonstrated an excellent degree of agreement between callipers and intraoral scanners using an inter-method intraclass correlation coefficient ranging from 0.904 to 0.999.

Since CBCT was first applied in the field of dentistry in the 1990s, it can almost accurately assess the characteristics of soft and hard tissues without obvious magnification and distortion and is increasingly used by clinicians to assess the anatomical morphology of teeth (29, 30). The intraclass correlation coefficient of the three measurement items of 50 maxillary first premolars between callipers and CBCT software were pretty high (mesiodistal diameter: 0.959, buccolingual diameter: 0.997, crown length: 0.999). This is similar to the study by Schwindling et al. (31), where the results indicated that the CBCT methods have a high degree of accuracy in predicting the mesiodistal diameter of a tooth. Gahleitner et al. (32) also detected a high degree of agreement between CBCT software measurement and calliper measurement by analysing the results of a paired *t*-test and regression analysis of the measurement of the mesiodistal and buccolingual diameters of 101 extracted impacted teeth.

Given that the consistence of both intraoral scanner measurements [*R*^2^: 0.726–0.986 (except for the crown length of UC)] and CBCT software measurements (*R*^2^: 0.920–0.998) with calliper measurement has been proven, the 308 extracted primary teeth and CBCT images of 407 primary teeth were pooled into the total sample (*N *= 715). The analysis of the total sample and reference to the previous literature about morphology data of primary teeth from other populations can be seen in Tables 4–12. The measurement results of the buccolingual diameter of the total sample in this study were compared with the corresponding measurement results of the other five populations, and the crown index and crown area were compared with the corresponding measurement results of the other three populations.

After considering the above four measurement items, a conclusion can be drawn that the primary teeth of northwest Chinese are comparatively larger than the primary teeth of other populations (Japanese, American white, African American, Icelander, Spanish, and Dominican Mulatto) but comparatively smaller than the primary teeth of the Australian Aboriginal population. When compared with the primary teeth of the Indian population, the primary teeth of northwest Chinese tend to be larger in mesiodistal diameter and smaller in buccolingual diameter. The difference between the anatomical morphology size of primary teeth measured in this study and the results of different populations can be caused by different genetic backgrounds and environmental factors. Just as the research of Hughes et al. (33) showed, similar to permanent teeth, variations in the primary crown size could be explained adequately by additive genetic and unique environmental components of variance.

Compared with the results of the study by Wang Huiyun, the mesiodistal and buccolingual diameters and crown length of the present total sample for all the primary teeth type were significantly smaller at the 0.1% level, except for the mesiodistal diameter of UD and LE and crown length of LB, which were significantly smaller at the 1% level. This is not consistent with the results of the study by Gadsbøll et al. (24), which found that the size of primary teeth was slightly larger than that of 50 years ago in North American white children. However our study is consistent with what McCollum and Sharpe (34) pointed out in the 1970s that the reduction in the size of the jaws during hominid evolution was accompanied by a general reduction of tooth size. It may also be related to the fact that the samples measured by Professor Wang Huiyun were all from East China, and there is a lack of a large-scale anatomical database of children's deciduous teeth from different regions of China.

Sexual dimorphism generally refers to the inherent and obvious differences between the sexes in some variables, reflecting the structure and functional characteristics of the body in heterogeneous sexual organisms, based on which people can judge the sex of an individual (35). The results of the present study indicated that the primary teeth of males are usually larger than those of females. Just as Gahleitner et al. (32) pointed out, gender differences may also need to be considered when conducting treatment and product development related to the restoration of dental defects in paediatric dentistry. In the field of dentistry, most research on sex dimorphism focused on permanent teeth, while the sex dimorphism research on deciduous teeth in the literature only involved the mesiodistal and buccolingual diameters (35). Our study found that the sex dimorphism percentage for the mesiodistal diameter of the primary teeth of northwest Chinese ranged from 0.59 to 2.54, which is similar to previous literature (35–37), while the sex dimorphism percentage for the buccolingual diameter ranged from 0.80 to 2.74, which is larger than previous literature (35). Moreover, the sex dimorphism percentage for the mesiodistal and buccolingual diameters of the primary teeth of northwest Chinese were both much smaller than that the sex dimorphism percentage of permanent teeth reported in the literature (38). Additionally, our study did not find the canine “field” of sexual dimorphism proposed by Garn et al. (38) in 1967, which suggested that teeth adjacent to the canine have a tendency for a greater percentage of dimorphism—the greater the canine dimorphism, the greater the sexual dimorphism of adjacent teeth.

This study has several limitations. There were few studies on the crown length of deciduous teeth, and only some studies (8) measured the crown length of the clinical crown, while this study measured the crown length of the anatomical crown. Therefore, the measurement results of this study cannot be compared with the results of previous studies. Moreover, as the data of other populations used for comparison in this study were all from results of previous studies, we could not normalise the baseline data of these populations. Because the manpower, material resources, and funding for this study cannot be performed in a multicentre study, it may make the results less extrapolated. However, in subsequent studies, we will slowly incorporate more analyses from other regions to make the results more refined and representative.

In summary, the morphological data of the primary teeth of children collected in this study can provide some important references for dental restorative treatment, growth and development research, crowding prediction, and the treatment of malocclusion in clinical practice. This includes the genetic background and environmental factors causing differences in the anatomical shape and size of the primary teeth between different populations, which can be considered from the environmental and genetic perspectives when treating and correcting the teeth to adopt a more comprehensive treatment approach. The primary teeth of males are usually larger than those of females, and this can provide a reference for the clinical judgment of gender. Additionally, this study concluded that native teeth showed good agreement in the central axis diameter using callipers and intraoral scanners, and the CBCT method had high accuracy when predicting the central axis diameter of the tooth. Therefore, intraoral scanners and CBCT can be used to measure and predict teeth with good accuracy and convenience in clinical practice.

## Data Availability

The original contributions presented in the study are included in the article/Supplementary Material, further inquiries can be directed to the corresponding author.
